# Science and Socialism

**Published:** 1887-10-15

**Authors:** 


### The Hospital

A Weekly Institutional Journal of

### Science, Medicine, and Philanthropy.

*For Hospitals, Asylums, Medical Practitioners, Students, Nurses, Families, Parishes, Congregations, and the Charitable Public.*

Vol. III. No. 55.] [October 15, 1887.

### Science and Socialism.

The Church Congress sitting at Wolverhampton last week gave voluntary and sympathetic audience to Mr. W. H. Champion, one of the apostles of Socialism. The members of the Congress thereby showed how far they have advanced in the ways of science and in the application of her modern methods. The prime essential of scientific progress is the patient interrogation of facts. Guesses, rough-and-ready processes, *per saltum* conclusions, are at a discount in the market of science. No honest broker will have anything to do with them; they are the marked cards of the speculator and the gambler. The clergy undertake to deal with the moral and spiritual ills of mankind, just as the doctor undertakes those of the physical organism. How does the physician set about his work? Does he prescribe his nostrum first of all, and then examine his patient afterwards, or not at all? Too often the preacher, the politician, or the "saviour of society" never condescends to any thorough study of the special case he has to deal with, but elaborates a detail of some antiquated metaphysic, ethic, or political system in his privacy, and then, like the vendor of quack pills, proclaims it as the universal remedy for every evil mankind is called upon to endure. But how does the physician set about his work at the bedside? Taking no preconceived opinions with him, he strips his patient; examines him in every region of the body; in every separate system; in every part of his personal and family history; in every physiological process. No detail is too minute; no symptom too insignificant; no pain too trifling; no fact in the history too remote for his large and comprehensive survey. Having made his examination he gives his judgment; or it may be he suspends his judgment, waiting for further investigation or for more careful thought. Then, and not till then, he prescribes his treatment. The treatment is absolutely determined, not by preconceived notions, but by the nature of the case. If the case be doubtful and obscure, the treatment is temporary and expectant; if it be clear beyond all doubt, a well-considered plan is thought out, and the first steps are taken with decision and promptitude.

The example of the physician is chosen because it illustrates simply and well the methods of modern science in every field of its operations. It is here affirmed that these are the only methods which are of real avail in every region of human activity. Until these methods are universally applied progress is impossible, and confusion must reign supreme. Take the case of Socialism! The mere mention of the word is enough to put some people in a rage. They will not hear a word about it with patience. They hate its disciples, and would stamp them out of existence as they would stamp out a fire or knock a venomous serpent on the head. Now such people are ten times more dangerous than the Socialists; and if they attempted to carry their theories into practice society would be under the necessity of locking them up in lunatic asylums.

The Church Congress has done itself infinite honour by giving audience to Mr. Champion. He is a peculiar phenomenon in the social organism. Many of the clergy, no doubt, look upon him as morally diseased; some will even regard his system as a cancer. But even if their judgment be correct, what is the duty of the physicians of the body social? Is it the usual method for the physician to cure the disease by killing the patient? Does he not rather handle him with the utmost gentleness and care, and do his best—it may be by months of tedious watching—to remove the disease and restore the patient to health? We have often said in these pages, and we repeat it here with emphasis, that the *methods* of science are of more importance to men and women than the *facts* of science; and we are convinced that if those methods could be universally applied, many of the ills which afflict the community would gradually and entirely cease. The rough-and-ready people, the stampers out, the drivers of the Juggernaut cars that go on grinding everything which comes in their way, are the curse of every age, the most dangerous enemies of mankind.

What is Socialism in its essence? It is merely an attempt to secure for every man a perennial supply of bread and butter, a sufficient suit of clothing, and a bed on which he can be sure of sound and refreshing sleep. Nothing more. And what is the origin of it? A hungry stomach and an empty pocket, coats out at elbows, and no blankets for the winter nights. Is it strange that men and women, being reasoning animals, and finding themselves assailed by cold and hunger, should think out some method of putting such urgent enemies to flight? And is it surprising that these hungry ones, being for the most part uneducated, or at least half educated, their schemes should be destitute of the academic stamp; nay, that they should even sometimes involve more or less serious inroads upon the ten commandments? Many of the Socialists are hungry, and, as the Yorkshire proverb says, "a hungry man is an angry man"; and an angry man is often an unreasonable man, and sometimes even a wicked man; nay, he has been known to be even a murderous man. But if the Socialist, in the extremity of his hunger and anger, talks foolishly of taking by force that which does not belong to him, is that a reason for at once dispensing with judge and jury and knocking him on the head? It is a reason for taking some measures to put money into his pocket, food into his stomach, and sense into his brain. The sense, indeed, may be trusted to enter of itself if the pocket and the stomach are satisfied. It is an axiom in medicine, "Remove the cause and the effects will pass away."

Granted that Socialism is a disease, that it is even of the nature of a serious epidemic of dangerous fever, we must consider how it is to be rationally dealt with. Panic is the last thing to be rushed into. How does the medical profession deal with epidemics? When the lower animals become the subjects of dangerous infectious disorders, as cows of foot-and-mouth disease, and dogs of rabies, the stamping-out course is adopted—we prevent the spread of infection by killing the patients. But do we ever adopt that method with man? If half London were in scarlet-fever hospitals to-morrow, where is the madman who would propose to the other half to kill them in order to stop the epidemic? And if in a case where all our lives would be at stake reason would still prevail, and the attempts made to check the disease could only be such as were consistent with the safety of the patient, surely in the case where property only is threatened, and that is in no danger because of the number and strength of its protectors—surely, we say, Socialism also may very well be dealt with by such methods of patient investigation and rational treatment as are applicable to every other social difficulty and danger. Steady work and good wages would convert nine-tenths of the Socialists into sound Conservatives or moderate Liberals in six months.

The winter is now before us. Trade is not prosperous, and there is a prospect of much destitution. That destitution is bound to rouse angry feelings in the hearts of the sufferers and their sympathisers. There are those who will be ready with their coarse and vulgar nostrums, which are the exact counterpart of the unreasoning anger of the Socialists. But the right and the successful way is to adopt the methods of science. Let the grievances of the poor be looked fairly in the face; wherever it is possible let the causes be removed; where that is not possible let substantial help be forthcoming until the difficult times are past; let emigration schemes be put forward, and, if necessary, public works initiated. These methods may involve the expenditure of money and some diminution of the resources of the prosperous. But what would the rougher methods involve? The spending of much more money, a far greater loss to the wealthy, and a result which would be disastrous both to the poor and the rich. When the reasonable methods of scientific investigation and brotherly help are powerless to check revolution in a State, that State is hastening to its doom.

